# METTL7B Expression in Sepsis: Association With Disease Severity and 28‐Day Mortality

**DOI:** 10.1002/jcla.70366

**Published:** 2026-09-28

**Authors:** Yuxuan Zhang, Yonghua Wang, Mengjiao Yang, Dan Huang

**Affiliations:** ^1^ Department of Critical Care Medicine The First Affiliated Hospital of Chengdu Medical College Chengdu Sichuan China; ^2^ Department of Emergency The Affiliated Chengdu 363 Hospital of Southwest Medical University Chengdu Sichuan China

**Keywords:** 28‐day mortality, combined prediction model, METTL7B, sepsis

## Abstract

**Background:**

Sepsis is a life‐threatening condition associated with high mortality, and current biomarkers remain suboptimal in prognostic evaluation. This study aimed to explore the associations of whole blood methyltransferase‐like 7B (METTL7B) expression with disease severity and 28‐day mortality in sepsis patients.

**Methods:**

In this prospective cohort study (93 sepsis patients, 22 non‐septic ICU controls), whole blood METTL7B (RT‐qPCR) and conventional biomarkers (SOFA, PCT, CRP, IL‐6) were measured. Dynamic SOFA changes (ΔSOFA from admission to Day 7) were assessed. ROC curves, logistic regression, and a combined model were used.

**Results:**

METTL7B levels were significantly elevated in sepsis patients compared to non‐septic controls (5.6 ± 1.33 vs. 2.44 ± 0.54, *p* < 0.001), with higher levels observed in non‐survivors. Baseline METTL7B levels were significantly higher in patients with a deteriorating SOFA trajectory (ΔSOFA ≥ +2) compared to those with stable or improving courses (*p* < 0.05). The AUC for METTL7B alone in predicting 28‐day mortality was 0.779, outperforming CRP and IL‐6. The combination of METTL7B with SOFA and PCT achieved the highest predictive accuracy (AUC = 0.854). Multivariate logistic regression identified METTL7B as associated with disease severity and 28‐day mortality risk in patients with sepsis (OR = 1.928, *p* = 0.017).

**Conclusion:**

Elevated whole blood METTL7B expression is associated with disease severity and unfavorable outcomes in septic patients. When combined with dynamic SOFA and PCT, it improves prognostic stratification. These findings suggest its potential value as a severity‐associated complementary marker warranting multi‐center validation.

## Graphical abstract

This prospective cohort study demonstrates that whole blood METTL7B expression is significantly elevated in sepsis patients upon ICU admission and independently associates with disease severity and 28‐day mortality. METTL7B alone predicted 28‐day mortality with an AUC of 0.779, and its combination with dynamic SOFA score and PCT achieved the highest predictive accuracy (AUC = 0.854). These findings position METTL7B as a severity‐associated, complementary prognostic biomarker in sepsis, warranting further validation in larger multicenter studies.

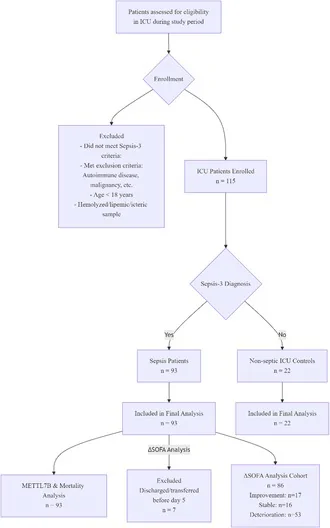

## 1 Introduction

Sepsis is a common and complex clinical syndrome characterized by a dysregulated host response to infection, leading to life‐threatening organ dysfunction. According to the Sepsis‐3 consensus definition issued in 2016, sepsis is now defined as “life‐threatening organ dysfunction caused by a dysregulated host response to infection” [1]. Data from the Global Burden of Disease Study indicate that sepsis is responsible for approximately 19.7% of all global deaths, with an age‐standardized mortality rate of 148.1 per 100,000 population. Sepsis has thus been recognized as a major global public health concern and imposes a significant economic burden on healthcare systems [2]. Despite advances in medical research and improvements in clinical management over the past three decades, sepsis remains a major cause of mortality [3]. Therefore, the identification of novel biomarkers for early prognosis and risk stratification is needed to guide timely therapeutic interventions and improve patient outcomes.

These limitations underscore the ongoing need for novel molecular biomarkers. Methyltransferase‐like 7B (METTL7B) is an S‐adenosylmethionine (SAM)‐dependent RNA methyltransferase involved in the epigenetic regulation of inflammatory gene expression and mitochondrial function. It plays key roles in lipid metabolism disorders, cancer progression, and infectious inflammation [4, 5]. Our previous studies have shown that METTL7B is upregulated in peripheral blood mononuclear cells (PBMCs) of sepsis patients. In vitro experiments further revealed that modulation of METTL7B expression affects lipopolysaccharide (LPS)‐induced cytokine production and macrophage polarization, implicating it in the immunopathogenesis of sepsis [6]. These observations led us to hypothesize that METTL7B might be associated with clinical outcomes in sepsis, meriting further investigation as a candidate prognostic marker.

Currently, the clinical evaluation of sepsis primarily relies on the Sequential Organ Failure Assessment (SOFA) score and several inflammatory biomarkers such as procalcitonin (PCT), C‐reactive protein (CRP), white blood cell count (WBC), and interleukin‐6 (IL‐6) [7, 8]. While these indicators are widely used for diagnosis, disease monitoring, and prognosis assessment, their individual predictive power is limited due to the heterogeneity and complexity of sepsis pathophysiology [9, 10]. For example, CRP and PCT may be elevated in non‐infectious inflammatory conditions, while IL‐6, despite its direct role in inflammatory cascades, has a short half‐life that complicates its clinical use for continuous systemic monitoring [11, 12]. Thus, the discovery of novel biomarkers that can complement existing tools and improve the accuracy of prognosis prediction is of critical clinical significance. Therefore, this study aimed to investigate the prognostic value of METTL7B for 28‐day mortality in sepsis using a prospective cohort design. We sought to compare its predictive capacity with conventional biomarkers and explore its potential contribution to integrated prognostic models. The findings from this exploratory research may offer insights for refining risk assessment and provide a basis for further validation of METTL7B in sepsis management.

## 2 Materials and Methods

### 2.1 Study Population and Clinical Parameters

This prospective cohort study included 115 patients admitted to the Department of Critical Care Medicine at the First Affiliated Hospital of Chengdu Medical College between January and December 2024. Among them, 93 adult patients met the diagnostic criteria for sepsis according to the Sepsis‐3 definition and were enrolled in the sepsis group. The control group consisted of 22 age‐ and sex‐matched non‐septic ICU patients admitted for conditions such as respiratory failure (*n* = 9), altered consciousness (*n* = 5), other types of shock (*n* = 5), and acute decompensated heart failure (*n* = 3), rather than undergoing stable, chronic management. All participants were followed for 28 days, and survival status at ICU discharge or death was recorded. Clinical data collected included SOFA score, WBC count, PCT, CRP, IL‐6, site of infection, and 28‐day survival status. This study was approved by the Ethics Committee of the First Affiliated Hospital of Chengdu Medical College (Approval No. 2022CYFYIRB‐SQ‐28). All methods were performed in accordance with the relevant guidelines and regulations and in compliance with the Declaration of Helsinki. Written informed consent was obtained from all participants or their legal guardians prior to enrollment.

### 2.2 Inclusion and Exclusion Criteria

Inclusion criteria:

1. Age ≥ 18 years
2. Admission to ICU from the general ward or emergency department due to life‐threatening conditions (e.g., shock, respiratory failure, high‐risk surgery, renal failure, or heart failure)
3. Diagnosis of sepsis defined as a SOFA score ≥ 2 and suspected infection based on the Sepsis‐3 definition [13].

Exclusion criteria:

1. Patients with autoimmune diseases, coronary artery disease, atrial fibrillation, chronic gastrointestinal disorders, AIDS, malignancies, or those who had used immunosuppressants within the previous 2 weeks [14].
2. Patients were transferred between ICUs during the study period.
3. Incomplete clinical data, laboratory results, or follow‐up information
4. Whole blood samples showing hemolysis (absorbance at 414 nm < 0.2), lipemia (triglycerides ≥ 400 mg/dL), or jaundice (bilirubin ≥ 20 μmol/L).

### 2.3 Outcome Measures

Primary outcome: 28‐day all‐cause mortality: Patients were stratified into survivor and non‐survivor groups based on 28‐day outcomes. METTL7B levels were compared between the two groups, and its predictive value was assessed.

Secondary outcomes:

1. Correlation between METTL7B levels and disease severity: Comparison of METTL7B expression in patients with SOFA score ≥ 8.5 versus < 8.5.
2. METTL7B expression in different infection sites: Comparison among patients with respiratory, abdominal, urinary tract, and intracranial infections.
3. Comparison of predictive performance between METTL7B and conventional inflammatory markers (WBC, CRP, PCT, IL‐6) using ROC analysis.
4. Evaluation of a combined predictive model incorporating METTL7B, SOFA, and PCT.

### 2.4 Sample Collection and RNA Quantification

Blood samples for METTL7B and other biomarker measurements were collected uniformly at the time of initial clinical assessment and ICU admission, prior to any specific ICU interventions beyond initial resuscitation. All blood samples were obtained before the administration of the first dose of broad‐spectrum antibiotics in the ICU. Take 3 mL of EDTA anticoagulated whole blood, centrifuge (2000 g, 10 min, 4°C) to separate the white blood cell layer, add TRIzol to fully lyse; add chloroform for centrifugal separation and layering, then transfer the aqueous phase, add an equal volume of isopropanol (containing glycogen for sedimentation) to precipitate RNA; after two washes with 75% ethanol, dissolve in RNase‐free water. RNA quality was verified via A260/A280 ratio (1.8–2.0) and RIN > 7.0 using an Agilent 2100 Bioanalyzer. cDNA synthesis was performed using the Prime Script RT reagent kit, and quantitative real‐time PCR (RT‐qPCR) was conducted using SYBR Green chemistry on a Bio‐Rad CFX96 system. GAPDH was selected based on previous studies evaluating METTL7B expression in blood‐derived samples and its commonly used application in RT‐qPCR normalization. Nevertheless, we acknowledge that reference gene stability may vary during systemic inflammation. Each sample was tested in triplicate, and relative METTL7B expression was calculated using the 2^−ΔΔCt method.

*Primer sequences*:

**Table:** 

Gene	Primer sequence (5′→3′)
METTL7B‐F	GCAACCGCAAGATGGAGAG
METTL7B‐R	GATTTGGGTCTAGGCAGGTGA
GAPDH‐F	CTGGGCTACACTGAGCACC
GAPDH‐R	AAGTGGTCGTTGAGGGCAATG

*Thermal cycling conditions* (*Bio‐Rad CFX96*):

Initial denaturation at 95°C for 30 s, followed by 40 cycles of 95°C for 5 s and 60°C for 30 s. Melting curve analysis was performed from 65°C to 95°C (0.5°C increment every 5 s).

### 2.5 Statistical Analysis

Firstly, the Shapiro–Wilk test was used to determine whether the measurement data followed a normal distribution. If the data met the normal distribution, *t*‐tests were employed to compare the differences between groups, and the results were presented as the mean ± standard deviation (Mean ± SD); for the data that did not follow a normal distribution, rank sum tests were used to compare the differences between groups, and the results were expressed as the median and interquartile range (Median & IQR). Categorical variables are presented as counts (percentages) and were compared using the Chi‐square test or Fisher's exact test if any expected cell count was less than 5. ROC curves were generated to evaluate the diagnostic performance of METTL7B and other biomarkers. Area under the curve (AUC), optimal cut‐off points, sensitivity, and specificity were calculated, and used Bootstrap resampling (1000 repetitions) to validate the AUC results; a difference was considered statistically significant if the 95% CI did not include zero [15]. Binary logistic regression was applied to identify predictors of 28‐day mortality. Statistical analyses and figure plotting were performed using GraphPad Prism version 8.0. *p* value < 0.05 was considered statistically significant.

## 3 Results

### 3.1 Baseline Characteristics of the Study Cohort

The enrollment process of the study participants is detailed in Figure 1. A total of 93 sepsis patients and 22 non‐septic ICU patients were enrolled. Baseline demographic and clinical data are summarized in Table 1. No significant differences were observed in age or sex distribution between groups. However, sepsis patients exhibited significantly higher SOFA scores (*p* < 0.001), along with elevated levels of WBC, CRP, PCT, and IL‐6. The predominant infection site among sepsis patients was the respiratory tract (60.9%), followed by intra‐abdominal (22.8%), intracranial (10.9%), and urinary tract (6.5%) infections. The 28‐day mortality was higher in the sepsis group than in controls (47.3% vs. 18.2%, *p* = 0.016).

**FIGURE 1:**
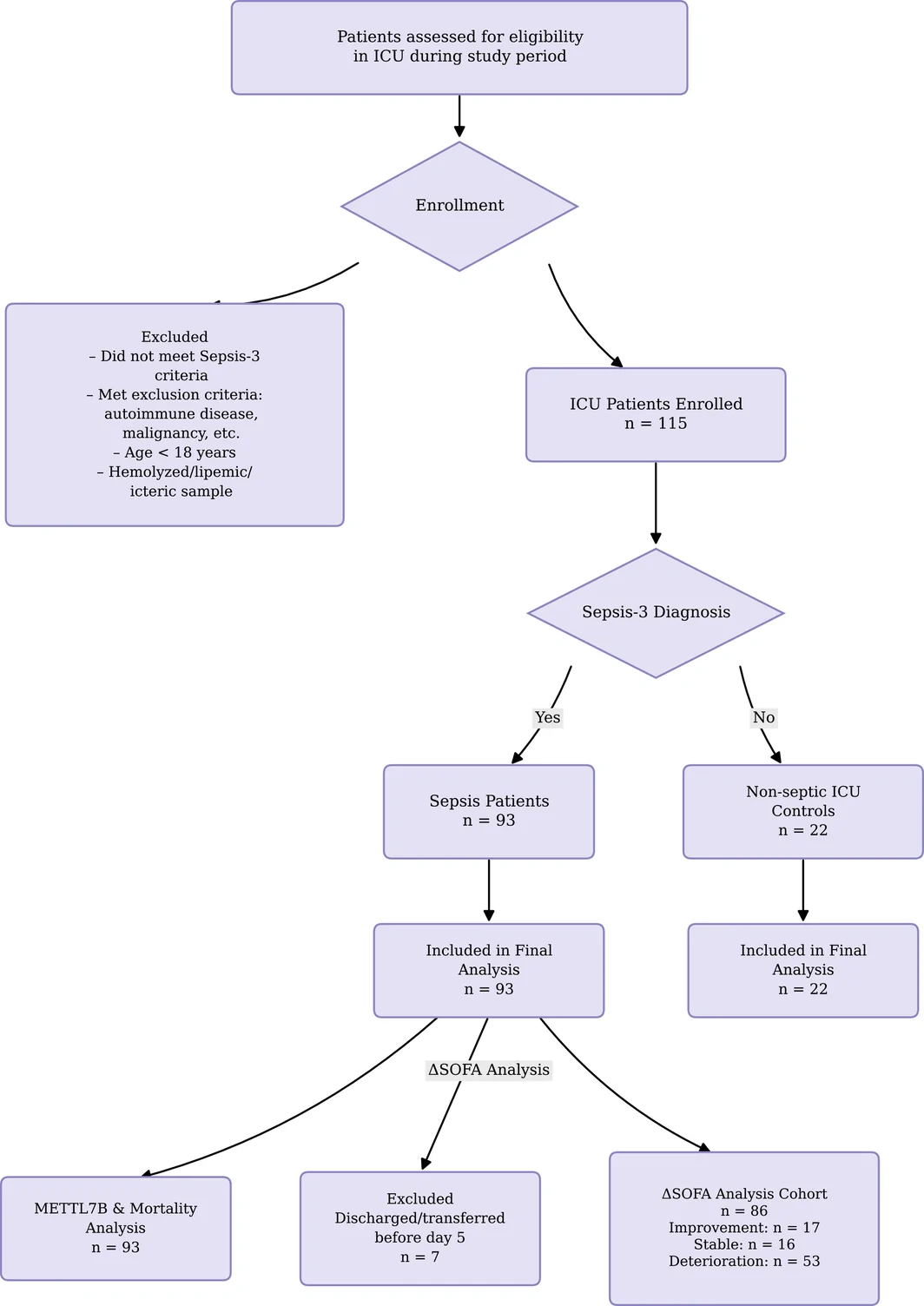
Patient enrollment and study flow diagram. Flowchart of patient selection and group allocation. The derivation of the sepsis cohort, non‐septic ICU control group, and the sub‐cohort for ΔSOFA analysis is shown.

**TABLE 1:** Baseline characteristics of sepsis patients and non‐septic ICU controls.

Characteristics	Sepsis patients (*n* = 93)	Non‐septic patients (*n* = 22)	*p*
Gender (Male %)	43 (46.2%)	13 (59.1%)	0.345
Age (years)	60 (53 ~ 68)	61 (52 ~ 71)	0.912
SOFA score	9 (6 ~ 13)	2 (1 ~ 3)	< 0.001
WBC (×10^9^/L)	13 (9.3 ~ 16.5)	6.85 (5.88 ~ 8.13)	< 0.001
CRP (mg/L)	180 (68 ~ 272)	11.5 (6.78 ~ 16.75)	< 0.001
PCT (ng/mL)	14 (2.65 ~ 46)	0.2 (0.08 ~ 0.4)	< 0.001
IL‐6 (pg/mL)	208 (101.5 ~ 425.5)	52 (22.5 ~ 90.75)	< 0.001
METTL7B	5.6 ± 1.33	2.44 ± 0.54	< 0.001
28‐day mortality (%)	44 (47.3%)	4 (18.2%)	0.016
Infection site			
Respiratory tract (%)	56 (60.9%)	—	
Intra‐abdominal (%)	21 (22.8%)	—	
Intracranial (%)	10 (10.9%)	—	
Urinary (%)	6 (6.5%)	—	

*Note:* Demographic, clinical, and laboratory parameters comparing sepsis patients (*n* = 93) and non‐septic ICU controls (*n* = 22). Normally distributed data (mean ± SD) were compared with the *t*‐test, and non‐normally distributed data (median, IQR) with the Mann–Whitney *U* test; categorical variables as counts (%).

Abbreviations: CRP, C‐reactive protein; METTL7B, relative mRNA expression (METTL7B/GAPDH); PCT, procalcitonin; SOFA, Sequential Organ Failure Assessment; WBC, white blood cell count.

### 3.2 Increased Whole Blood METTL7B in Sepsis and Its Association With Mortality

RT‐qPCR analysis revealed significantly elevated whole blood METTL7B expression in sepsis patients compared to non‐septic ICU controls (5.6 ± 1.33 vs. 2.44 ± 0.54, *p* < 0.001). Among sepsis patients, non‐survivors had markedly higher METTL7B levels than survivors (*p* < 0.001) (Figure 2). To further characterize the association between METTL7B and mortality, we compared baseline characteristics between 28‐day survivors (*n* = 49) and non‐survivors (*n* = 44) (Table S1). Non‐survivors had significantly higher baseline SOFA scores, PCT, and METTL7B levels (*p* < 0.001), while age, sex, CRP, IL‐6, WBC, and infection site distribution did not differ significantly between the two groups.

**FIGURE 2:**
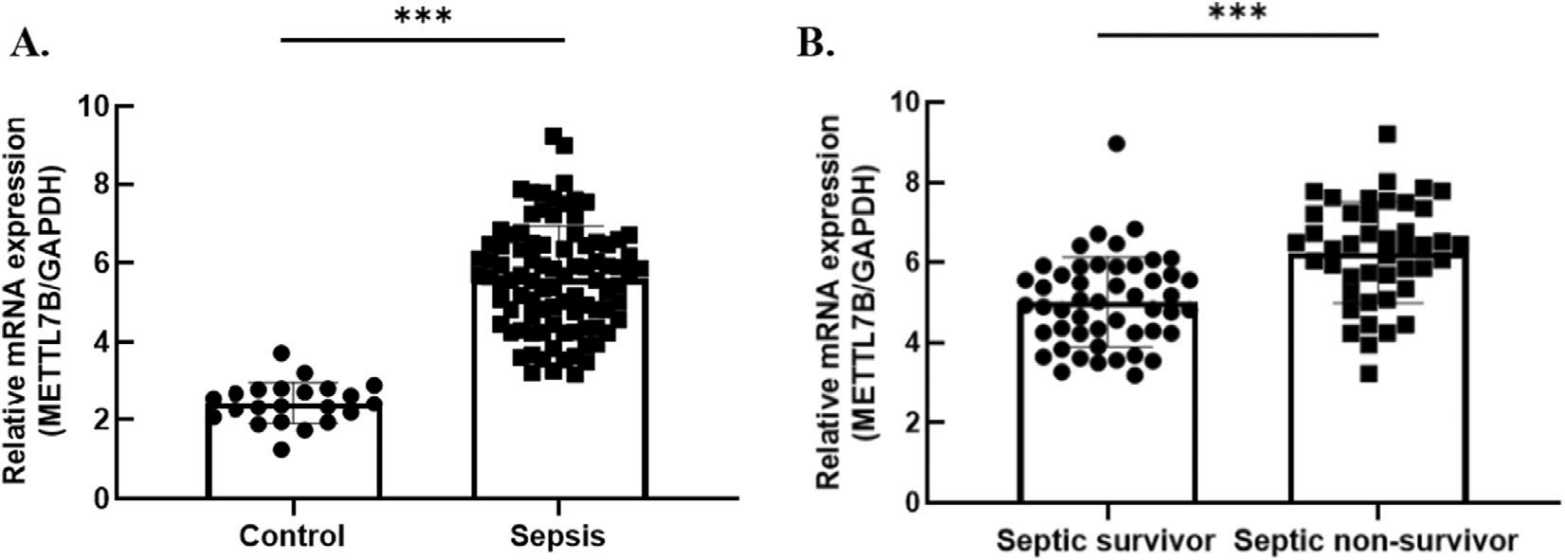
Whole blood METTL7B levels in sepsis patients and non‐septic controls. (A) METTL7B expression was significantly higher in sepsis patients (*n* = 93) compared to non‐septic ICU controls (*n* = 22). (B) Non‐survivors showed elevated METTL7B levels versus survivors at 28‐day follow‐up ***p < 0.001.

### 3.3 Differences and Correlation of METTL7B Concentrations With SOFA Scores in Sepsis

Stratification of sepsis patients based on SOFA scores revealed that those with SOFA ≥ 8.5 had significantly higher METTL7B levels than those with SOFA < 8.5 (*p* < 0.01) (Figure 3). To further characterize the association between METTL7B and mortality, univariable logistic regression analysis was performed, and the results are presented in Table S2. To assess the relationship between METTL7B expression and disease severity, we analyzed the baseline indicators of all 115 patients (including sepsis and non‐sepsis controls). Spearman rank correlation analysis revealed a highly significant positive correlation between the total blood METTL7B expression level and the baseline SOFA score (*r*_s_ = 0.665, *p* < 0.001), indicating that the METTL7B level systematically increased with the aggravation of organ dysfunction (Table S3).

**FIGURE 3:**
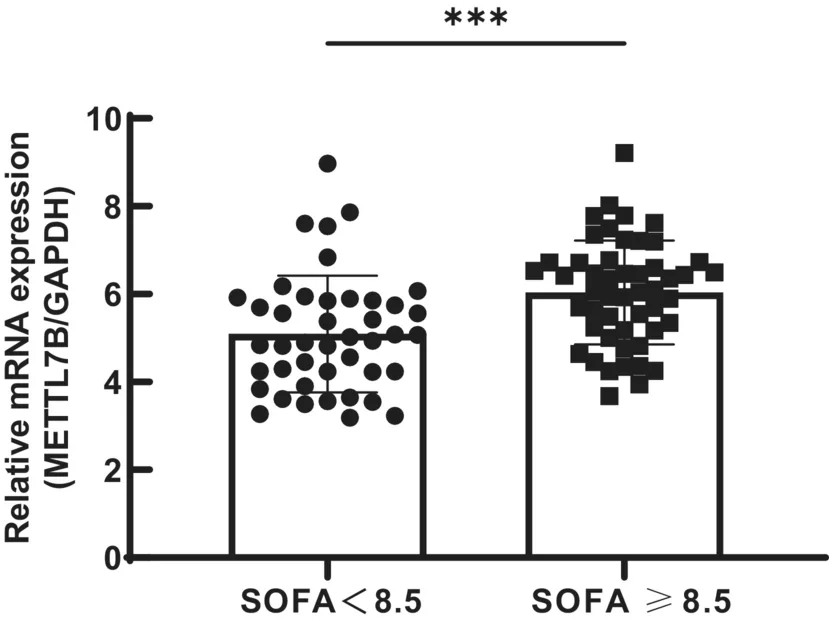
METTL7B expression stratified by SOFA score. Patients with SOFA ≥ 8.5 (*n* = 51) exhibited higher METTL7B levels than those with SOFA < 8.5 (*n* = 42) [16] ***p < 0.001.

### 3.4 Variation of METTL7B Expression Across Infection Sites

Among the 93 sepsis patients, those with intra‐abdominal (*n* = 21) and intracranial (*n* = 10) infections exhibited higher METTL7B levels compared to patients with respiratory (*n* = 56) or urinary tract (*n* = 6) infections. However, pairwise comparisons indicated that this overall difference was primarily driven by a significant elevation in METTL7B levels in patients with intracranial infections (*n* = 10) compared to those with respiratory tract infections (*n* = 56) (*p* < 0.05). No other pairwise comparisons (e.g., intra‐abdominal vs. respiratory, intra‐abdominal vs. intracranial) reached statistical significance (Figure 4).

**FIGURE 4:**
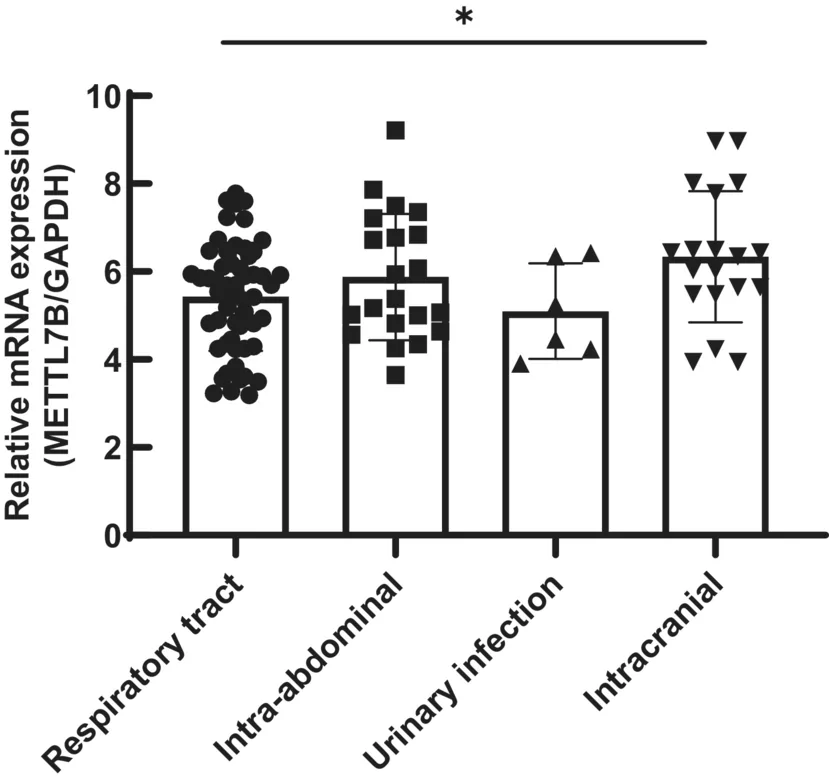
METTL7B levels across infection sites. Comparison of METTL7B expression in sepsis patients with different infection foci: Respiratory (*n* = 56), intra‐abdominal (*n* = 21), intracranial (*n* = 10), and urinary tract (*n* = 6) *p < 0.05.

### 3.5 Comparison With Conventional Inflammatory Markers

WBC, CRP, PCT, and IL‐6 levels were all significantly elevated in sepsis patients compared to controls. Among these, PCT showed a significant difference between survivors and non‐survivors (*p* < 0.05), while other markers were not significantly different between outcome groups (Figure 5).

**FIGURE 5:**
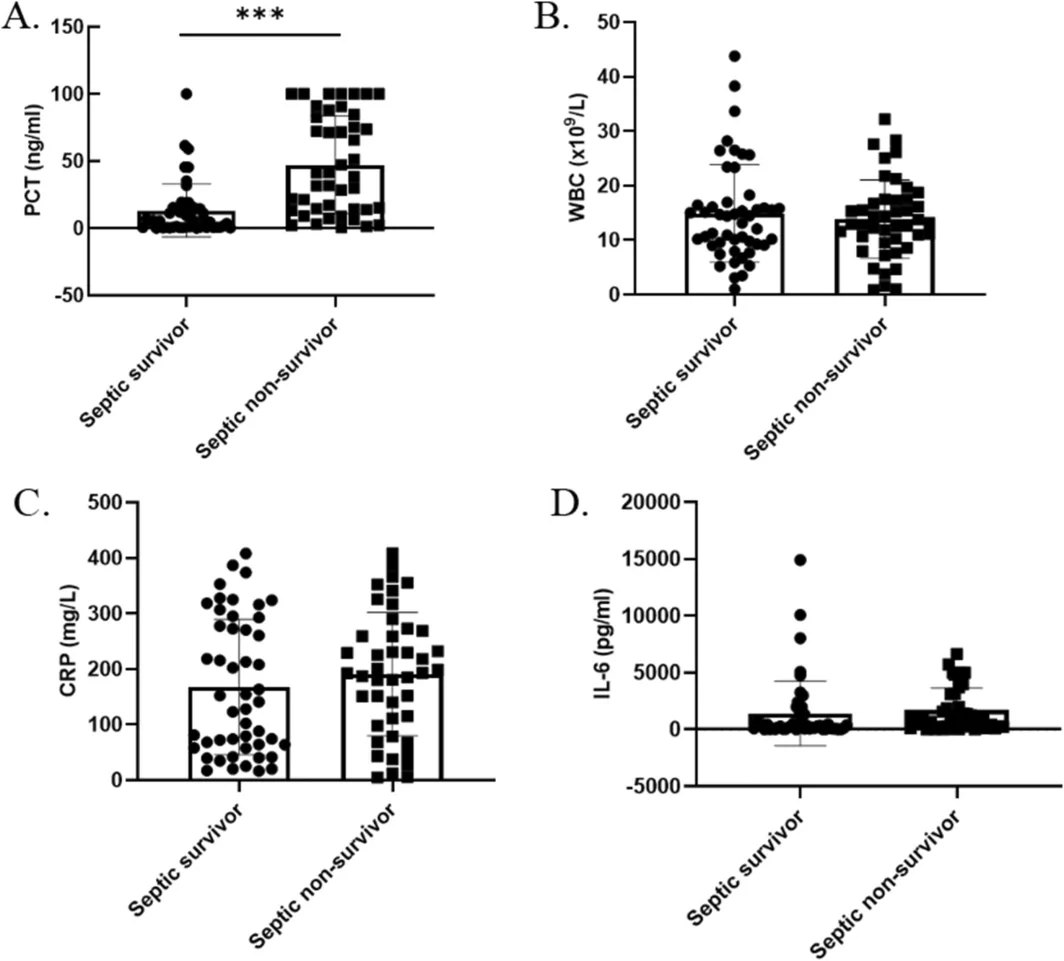
Conventional inflammatory markers in study cohorts. (A–D) Comparison of WBC, IL‐6, PCT, and CRP levels between sepsis patients and controls ***p < 0.001.

### 3.6 Dynamic SOFA Assessment and Association With METTL7B

The Sequential Organ Failure Assessment (SOFA) score was calculated at baseline (within 24 h of ICU admission) and reassessed on Day 7 (±1 day window) to analyze the dynamic organ dysfunction trajectory. Patients who were discharged or transferred to another ICU before Day 5 (*n* = 7) were excluded from the ΔSOFA analysis due to missing follow‐up SOFA data. Among the remaining 86 patients, those who died before Day 7 (*n* = 30) were classified as having a Deterioration trajectory from a clinical standpoint, as death represented the most severe outcome; however, they were not included in the ΔSOFA boxplot analysis (Figure 6) because Day‐7 SOFA scores were unavailable. For the 56 patients surviving to Day 7, ΔSOFA was calculated and they were stratified into three groups: Improvement (ΔSOFA ≤ −2, *n* = 17), Stable (ΔSOFA −1 to +1, *n* = 16), and Deterioration (ΔSOFA ≥ +2, *n* = 23). A sensitivity analysis was performed on this cohort of patients surviving to Day 7. Analysis of baseline METTL7B levels across these trajectories revealed a significantly higher level in the Deterioration group compared to the others (*p* < 0.05), indicating its value in predicting subsequent organ function decline. To further validate the robustness of this association, we performed a sensitivity analysis by including the 30 patients who died before Day 7 into the Deterioration group (Figure S3). The results remained consistent, with the Deterioration group (*n* = 53) still exhibiting significantly higher baseline METTL7B levels compared to the Improvement and Stable groups (*p* < 0.05), confirming that the observed association is not affected by the exclusion of early deaths.

**FIGURE 6:**
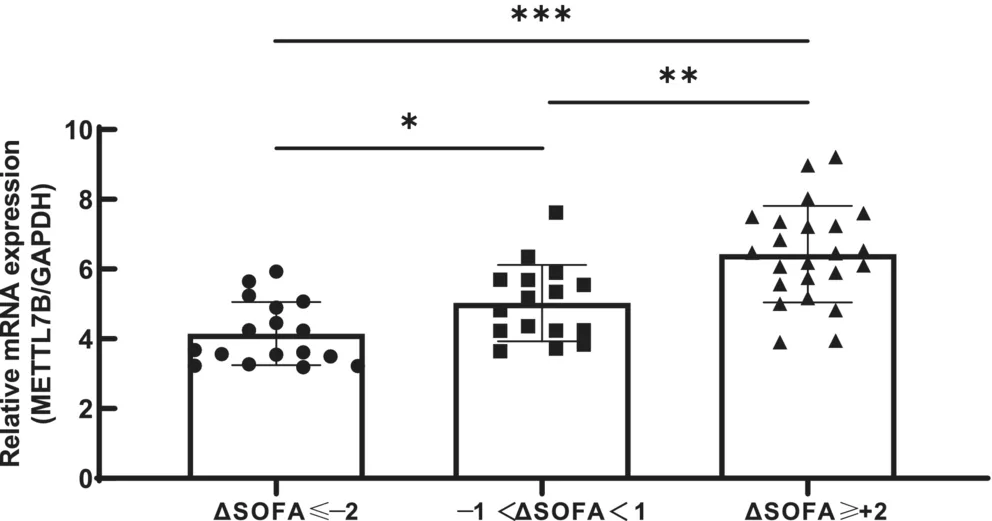
Association between baseline METTL7B levels and ΔSOFA. Only patients surviving to Day 7 were included in this analysis (*n* = 56); patients who died before Day 7 were not included because Day‐7 SOFA scores were unavailable. The central line in the box represents the median, the box represents the IQR, and the whiskers show the range *p < 0.05, **p < 0.01, ***p < 0.001.

### 3.7 Prognostic Value of METTL7B for 28‐Day Mortality

Among the 93 patients with sepsis, the 28‐day all‐cause mortality was 47.3%. Receiver operating characteristic (ROC) analysis was performed to evaluate the prognostic value of METTL7B and compare it with conventional biomarkers. The area under the curve (AUC) values for each parameter were as follows: METTL7B: 0.779 (95% CI: 0.683–0.875, *p* < 0.001), SOFA score: 0.797 (95% CI: 0.706–0.887, *p* < 0.001), PCT: 0.808 (95% CI: 0.721–0.895, *p* < 0.001), IL‐6: 0.618 (95% CI: 0.502–0.735, *p* = 0.05), CRP: 0.556 (95% CI: 0.437–0.674, *p* = 0.356), WBC: 0.501 (95% CI: 0.382–0.620, *p* = 0.991). These results indicate that METTL7B had better discriminatory ability than IL‐6, CRP, and WBC, and its predictive performance was comparable to that of SOFA and PCT (Figure 7, Table 2).

**FIGURE 7:**
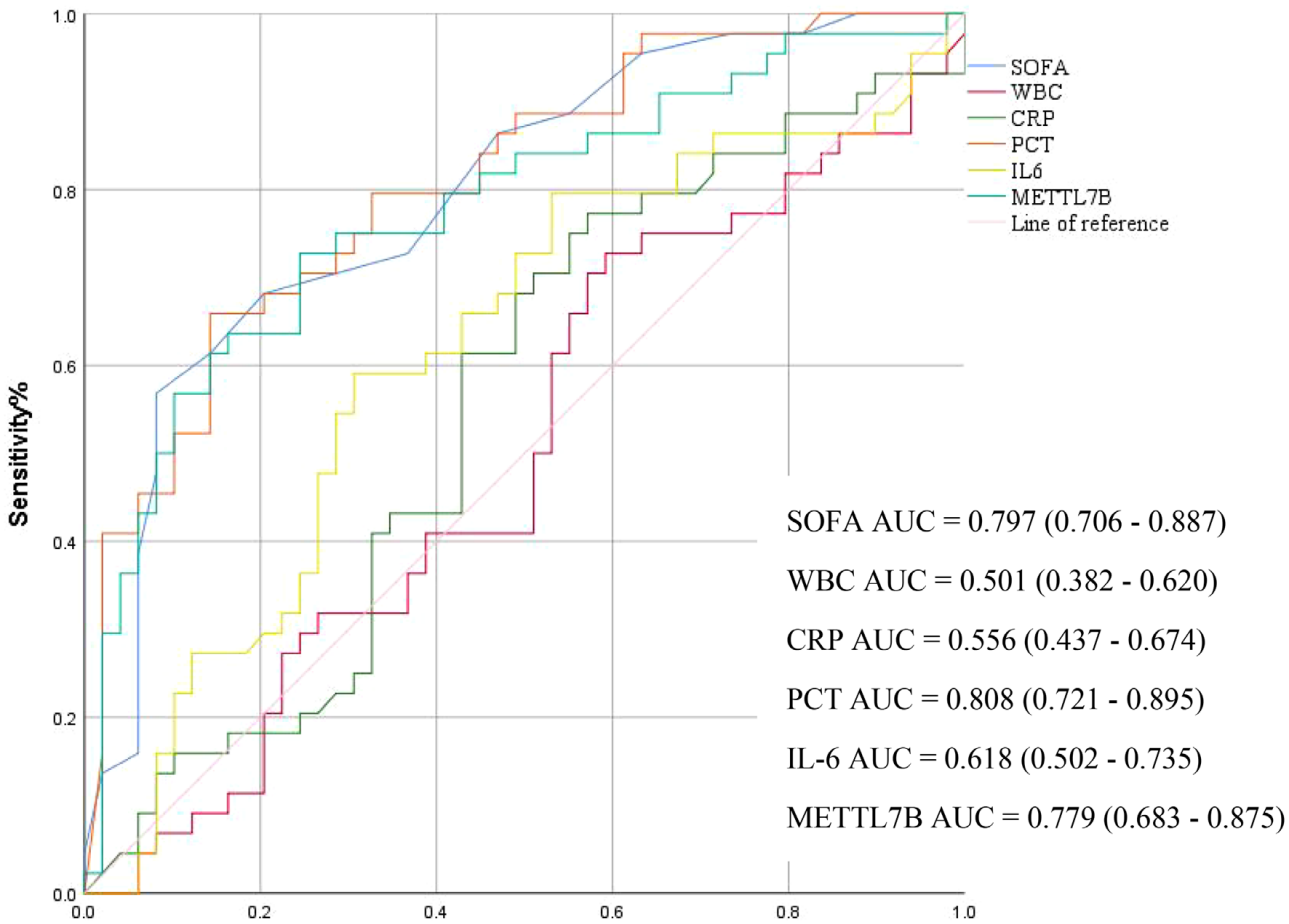
ROC analysis of mortality prediction. Receiver operating characteristic curves for individual biomarkers and their AUC comparisons. METTL7B (AUC = 0.779) showed superior predictive value to IL‐6 (0.618), CRP (0.556), and WBC (0.501), but was comparable to SOFA (0.797) and PCT (0.808).

**TABLE 2:** Predictive performance of biomarkers for 28‐day mortality.

Parameter	AUC (95% CI)	Cut‐off value	SE	SP	PPV	NPV
SOFA	0.797 (0.706–0.887)	11.5000	0.568	0.918	0.874	0.684
WBC	0.501 (0.382–0.620)	10.6600	0.727	0.408	0.545	0.601
CRP	0.556 (0.437–0.674)	93.0000	0.773	0.429	0.576	0.655
PCT	0.808 (0.721–0.895)	20.3400	0.659	0.857	0.824	0.72
IL6	0.618 (0.502–0.735)	599.5500	0.591	0.694	0.658	0.629
METTL7B	0.779 (0.683–0.875)	5.6949	0.727	0.755	0.745	0.735

*Note:* Receiver operating characteristic (ROC) analysis of METTL7B and conventional biomarkers. Optimal cut‐off values determined by Youden's index.

Abbreviations: AUC, area under the curve; CI, confidence interval; NPV, negative predictive value; PPV, positive predictive value; SE, sensitivity; SP, specificity.

### 3.8 Combined Predictive Models Incorporating METTL7B

To further explore the value of METTL7B in predicting 28‐day mortality in sepsis patients, we combined METTL7B with SOFA and PCT to assess 28‐day fatality. Results showed that the AUC of SOFA combined with PCT was 0.835 (*p* < 0.001, 95% CI: 0.753–0.918). When METTL7B was combined with SOFA, the AUC increased from 0.779 to 0.835 (*p* < 0.001, 95% CI: 0.754–0.917). The AUC of PCT combined with METTL7B was 0.833 (*p* < 0.001, 95% CI: 0.752–0.915), which was lower than that of the SOFA‐combined regimen. The highest AUC was observed in the three‐index combination, reaching 0.854 (*p* < 0.001, 95% CI: 0.7799–0.930). The combination of SOFA, PCT, and METTL7B yielded the highest AUC value (0.854) (Figure 8). Through Bootstrap resampling verification, a three‐indicator model integrating organ function (SOFA), systemic infection (PCT), and immune metabolism dimension (METTL7B) was combined, demonstrating the most stable discriminative performance (ΔAUC = 0.3749 (95% CI: 0.2702–0.4749)). This confirms that the combined use of these three indicators is superior to any combination of two indicators. It ensures the stability of the zero distribution and confidence intervals, reduces the influence of random errors on the conclusion, and indicates that the results have high robustness (Figures S1 and S2, Table S4).

**FIGURE 8:**
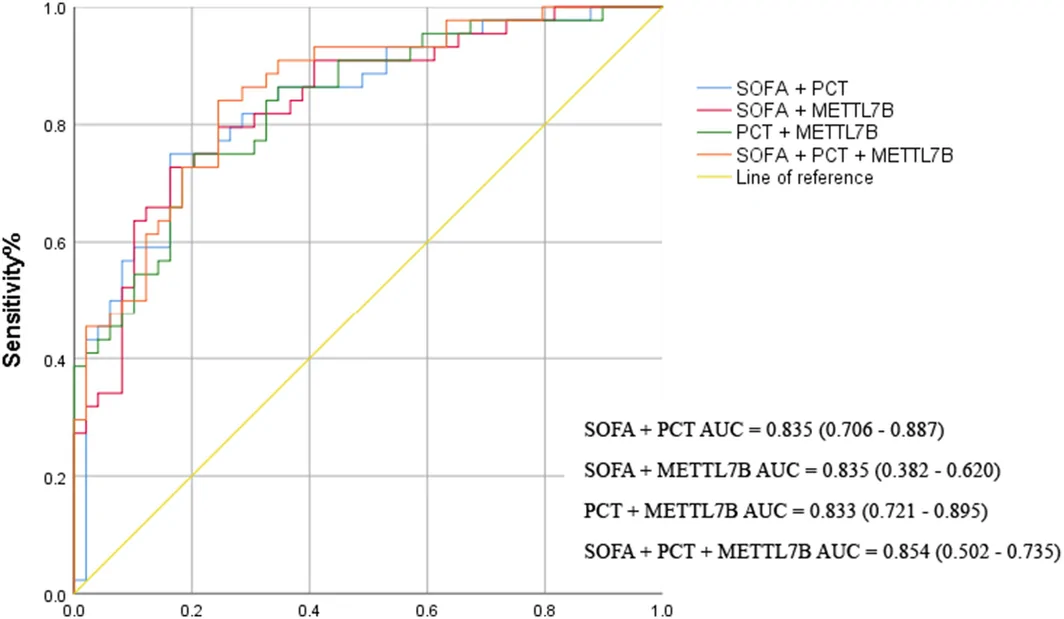
Combined predictive models. ROC curves of combination models: SOFA + METTL7B (AUC = 0.835), PCT + METTL7B (0.833), and triple combination (SOFA + PCT + METTL7B, AUC = 0.854). AUC comparison demonstrates synergistic improvement in predictive performance.

### 3.9 Novel Predictors of 28‐Day Mortality

Binary logistic regression analysis revealed that METTL7B (*B* = 0.656, OR = 1.928, *p* = 0.017), PCT (*B* = 0.025, OR = 1.026, *p* = 0.04), and SOFA score (*B* = 0.222, OR = 1.248, *p* = 0.018) on the day of ICU admission were predictors of 28‐day mortality in sepsis patients, whereas WBC, CRP, IL‐6, and infection site were not (Table 3).

**TABLE 3:** Multivariate logistic regression analysis of 28‐day mortality predictors.

Parameter	*B*	Standard error	Wald	OR	95% confidence interval	*p*
SOFA	0.222	0.094	5.621	1.248	1.039–1.5	0.018
WBC	0.055	0.039	1.970	0.946	0.876–1.022	0.160
CRP	0.002	0.003	0.620	0.998	0.993–1.003	0.431
PCT	0.025	0.012	4.220	1.026	1.001–1.051	0.040
IL6	0.001	0.001	1.365	1.000	1.001–1.003	0.243
METTL7B	0.656	0.276	5.648	1.928	1.122–3.312	0.017
Infection site	0.015	0.312	0.002	1.015	0.551–1.87	0.962

*Note:* Adjusted odds ratios (OR) with 95% confidence intervals (CI) for clinical parameters. Variables with *p* < 0.1 in univariate analysis (Table S2) were included in the multivariate model.

Abbreviations: CRP, C‐reactive protein; PCT, procalcitonin; SOFA, Sequential Organ Failure Assessment; WBC, white blood cell count.

## 4 Discussion

In this prospective cohort study, we investigated the association between whole blood METTL7B expression and 28‐day outcomes in patients with sepsis admitted to the ICU. Our main findings can be summarized as follows: (1) METTL7B expression was significantly elevated in patients with sepsis compared with non‐septic ICU controls, suggesting a potential association with critical inflammatory states; (2) METTL7B shows promising prognostic performance compared to traditional inflammatory markers such as WBC, CRP, and IL‐6; (3) METTL7B level was associated with subsequent organ dysfunction, being higher in patients with a deteriorating SOFA trajectory (ΔSOFA ≥ +2) than in those with stable or improving courses; (4) A combined predictive model incorporating METTL7B, SOFA, and PCT yielded the highest accuracy in our analysis (AUC = 0.854). While its absolute improvement over the SOFA + PCT model (AUC = 0.835) is modest, integrating this novel biomarker holds biological and translational promise; (5) METTL7B expression is associated with increased risk of 28‐day mortality in sepsis patients. The primary objective of this study was to explore its potential role as a severity‐ and prognosis‐associated molecular marker within an already diagnosed sepsis population. These results provide novel exploratory evidence that supports further investigation of METTL7B as a potential molecular target for risk stratification and sheds light on the immunometabolic mechanisms in sepsis.

Our data align with previous findings from our laboratory, where METTL7B was shown to be upregulated in peripheral blood mononuclear cells (PBMCs) from septic patients. Functional experiments have confirmed that METTL7B modulates LPS‐induced cytokine production and macrophage polarization, indicating its role in the immune response during sepsis [6]. Given the relatively complex isolation procedure and higher cost associated with PBMC extraction, this study utilized whole blood‐derived RNA from patients, which offers greater clinical translatability due to its convenient sampling and cost‐effectiveness. Similar findings have been observed in neonatal sepsis, where METTL7B expression was elevated and correlated with immune cell infiltration, including B cells, T cells, monocytes, neutrophils, and macrophages [17]. These observations support METTL7B as a potential immune‐related biomarker across age groups. METTL7B, as an RNA methyltransferase, is also involved in m6A modifications and the regulation of cellular oxidative stress and immune activity. It has been implicated in several cancer types, particularly in relation to immune cell infiltration, extracellular matrix remodeling, and epithelial‐mesenchymal transition [18]. In lung adenocarcinoma, METTL7B overexpression enhances m6A modification and contributes to treatment resistance [4]. In hepatocellular carcinoma, METTL7B regulates m6A‐dependent translation of key metabolic genes, promoting malignant phenotypes [19]. Recent studies have demonstrated that loss of m6A methylation contributes to cytokine storms and cardiac dysfunction in sepsis. For example, m6A methylation of Spi2a disrupts IKK complex formation, attenuating NF‐κB signaling and reducing inflammatory damage [20]. In sepsis‐associated acute lung injury, METTL3‐mediated m6A modification enhances ferroptosis through mitochondrial metabolic reprogramming [21]. Other research indicates that METTL7B suppresses histone deacetylase 3 (HDAC3) activity via ubiquitination, alleviating cardiac remodeling and improving function in heart failure models [22]. Furthermore, METTL7B has been found to regulate reactive oxygen species (ROS) generation, an important process in both cardiovascular disease and malignancy [23, 24]. Given that immune dysregulation and oxidative stress are central to the pathophysiology of sepsis [25, 26], we speculate that the sustained high expression of METTL7B in non‐survivors may be linked to mitochondrial dysfunction, oxidative stress, and epigenetic modifications, thereby exacerbating organ failure and energy metabolism imbalance. Our findings contribute to the conceptual framework of “sepsis–immunometabolic reprogramming–organ dysfunction” and provide clinical evidence for this theoretical axis.

Our study observed a difference in METTL7B expression levels related to infection sites in sepsis patients. Intracranial infections (*n* = 10) compared to those with respiratory tract infections (*n* = 56) (*p* < 0.05). These findings suggest that METTL7B may play distinct regulatory roles depending on the infection site. Of particular interest, METTL7B levels were markedly elevated in intracranial infections, likely due to the unique neuroimmune microenvironment shaped by the blood–brain barrier. Emerging evidence suggests that METTL7B may contribute to neuroinflammatory regulation. A recent study reported that Japanese encephalitis virus infection downregulates the expression of the m6A methyltransferase METTL3 in mouse brain tissues [27]. Although the exact role of METTL7B in this context remains unclear, our data imply its potential involvement in immune modulation during CNS infections. Previous studies have demonstrated that DNA methylation modulates the differentiation of regulatory T cells (Tregs), a critical mechanism in gut mucosal immune tolerance. For instance, FOXP3 methylation levels in colonic tissues show a significant positive correlation with the expression of DNMT1, DMAP1, METTL7B, and METTL10 [28, 29]. Thus, we hypothesize that the differential METTL7B expression observed in our study may reflect tissue‐specific immune regulation or variations in the host‐pathogen interaction microenvironment across different infection sites.

Importantly, METTL7B demonstrated prognostic value for adverse outcomes, with an AUC of 0.779 (95% CI, 0.683–0.875), sensitivity of 72.7%, and specificity of 75.5%. Within this cohort, its prognostic accuracy is comparable to that of PCT and appeared higher than that of CRP, IL‐6, and WBC [30, 31]. Moreover, the triple‐marker model (SOFA + PCT + METTL7B) achieved the highest AUC (0.854) in our analysis. Although the absolute increase over the SOFA + PCT model was modest, this finding illustrates the potential additive value of incorporating a novel molecular marker reflecting immunometabolic regulation (METTL7B) with established indicators of organ dysfunction (SOFA) and bacterial infection (PCT). The synergistic interaction of these parameters suggests that early risk stratification models could be enhanced by integrating diverse pathophysiological dimensions. This multi‐dimensional perspective could inform more nuanced assessment in complex ICU settings [32, 33, 34, 35, 36]. Our results, therefore, provide a methodological proof‐of‐concept for such integrative models and lay the groundwork for future validation studies in larger cohorts to assess their potential utility in supporting clinical risk stratification.

However, this study has several limitations. First, the single‐center design, relatively limited sample size, and the imbalance between groups, along with the lack of a healthy control group, make the conclusions drawn regarding the association between METTL7B and sepsis exploratory in nature. Second, the use of non‐septic ICU patients as controls, while controlling for some ICU‐related factors. Therefore, our findings should be interpreted as an association between METTL7B expression and septic severity/outcome rather than evidence of sepsis specificity. Future studies including healthy individuals and patients with other inflammatory diseases are required. Third, METTL7B was measured only at ICU admission using RT‐qPCR, a research technique not yet standardized for clinical use. The lack of serial measurements limits insights into its dynamic changes and causal role, while the single‐time‐point design cannot fully rule out confounding related to illness onset. Finally, the observed improvement in model performance with METTL7B, though promising, is exploratory and requires validation in larger, multicenter prospective studies with serial sampling to elucidate its clinical relevance and underlying mechanisms.

## 5 Conclusion

In conclusion, this prospective cohort study demonstrates that elevated whole blood METTL7B expression at ICU admission is associated with greater disease severity and increased 28‐day mortality in patients with sepsis. METTL7B shows prognostic value and provides information that appears complementary to established clinical indicators such as SOFA score and PCT, offering a novel multi‐dimensional tool for early risk stratification. METTL7B may represent a novel severity‐ and prognosis‐associated molecular marker in critically ill septic patients. While these findings highlight the potential of METTL7B to augment prognostic models and inform sepsis pathophysiology, they remain exploratory and thus require validation in larger, multicenter studies prior to any clinical application.

## Funding

This work was supported by the Sichuan Medical Association Fund (Grant No. Q21030).

## Conflicts of Interest

The authors declare no conflicts of interest.

## Supporting information


**Table S1:** Baseline characteristics of sepsis patients stratified by 28‐day survival status.
**Table S2:** Univariable Analysis of Factors Associated with Mortality.
**Table S3:** Correlation analysis of METTL7B expression and the severity of organ dysfunction.
**Table S4:** Bootstrap test the analysis results of each combination index.
**Figure S1:** Mean difference and 95% confidence interval.
**Figure S2:** Comparison of AUC values for each indicator combination.
**Figure S3:** Association between baseline METTL7B levels and ΔSOFA including patients who died before Day 7. Only patients surviving to Day 7 were included in this analysis (*n* = 56); patients who died before Day 7 were not included because Day‐7 SOFA scores were unavailable. The central line in the box represents the median, the box represents the IQR, and the whiskers show the range.

## Data Availability

The datasets analyzed during the current study are not publicly available due to ethical restrictions and patient privacy protection, but are available from the corresponding author on reasonable request.
