# Supplementary material for: METTL7B Expression in Sepsis: Association With Disease Severity and 28‐Day Mortality
Source: J Clin Lab Anal. 2026 Sep 28;40(21):e70366. doi: 10.1002/jcla.70366 (PMC13620335; doi:10.1002/jcla.70366)
Supplement: Supplementary file 1 [file JCLA-40-e70366-s001.docx]

Table S1: Baseline characteristics of sepsis patients stratified by 28-day survival status.

| Characteristics | Septic survivor (n = 49) | Septic non-survivor (n = 44) | P value |
| --- | --- | --- | --- |
| Gender (Male%) | 26(53.1%) | 24(54.5%) | 0.526 |
| Age (years) | 60.43 ± 8.99 | 60.52 ± 8.98 | 0.96 |
| SOFA score | 7(4 ~ 9) | 12(8 ~ 15) | < 0.001 |
| WBC (x10^9^/L) | 14.42(9.19 ~ 16.69) | 13.06(9.66 ~ 17.44) | 0.991 |
| CRP (mg/L) | 141.2(60.9 ~ 285.3) | 192.8(101.3 ~ 266.58) | 0.356 |
| PCT (ng/ml) | 5.93(0.75 ~ 14.98) | 39.82(13.99 ~84.19) | < 0.001 |
| IL-6 (pg/ml) | 262.3(73.25 ~ 1511) | 877.5(216.95 ~ 3132.45) | 0.052 |
| METTL7B | 4.93(4.24 ~ 5.79) | 6.4(5.42 ~7.23) | < 0.001 |
| Infection site | | | 0.178 |
| Respiratory tract(%) | 32 (65.3%) | 24 (54.5%) |  |
| Intra-abdominal(%) | 12 (24.5%) | 9 (20.5%) |  |
| Intracranial(%) | 3 (6.1%) | 3 (6.8%) |  |
| Urinary(%) | 2(4.1%) | 8(18.2%) |  |

Note: Baseline characteristics of sepsis patients stratified by 28-day survival status, comparing survivors (n = 49) and non-survivors (n = 44). Data are presented as median (IQR) or mean ± SD for continuous variables, and as counts (%) for categorical variables. Normally distributed data were compared using the t-test, non-normally distributed data using the Mann-Whitney U test, and categorical variables using the chi-square test.

Table S2: Univariable Analysis of Factors Associated with Mortality

| **Variable** | **OR (95% CI)** | **P value** |
| --- | --- | --- |
| Age | 1.03 (0.99-1.07) | 0.102 |
| Gender | 1.12 (0.52-2.41) | 0.769 |
| SOFA | 1.32 (1.19-1.47) | <0.001 |
| WBC | 1.02 (0.96-1.08) | 0.561 |
| CRP | 1.00 (0.99-1.01) | 0.432 |
| PCT | 1.02 (1.01-1.03) | <0.001 |
| IL6 | 1.00 (1.00-1.00) | 0.003 |
| METTL7B | 2.15 (1.52-3.04) | <0.001 |
| Infection Site: Abdominal (ref: Pneumonia) | 1.85 (0.68-5.05) | 0.229 |
| Infection Site: UTI (ref: Pneumonia) | 0.92 (0.19-4.39) | 0.917 |
| Infection Site: CNS (ref: Pneumonia) | 3.67 (1.18-11.43) | 0.025 |

Table S3: Correlation analysis of METTL7B expression and the severity of organ dysfunction

| **Parameter** | **r_s** | **P value** |
| --- | --- | --- |
| Baseline METTL7B vs. Baseline SOFA score | 0.665 | P＜0.001 |

Note: Spearman correlation analysis across all patients (N=115) shows a strong positive association (r_s~ = 0.665, p < 0.001) between baseline METTL7B expression and baseline SOFA score.

Table S4: Bootstrap test the analysis results of each combination index

| **Parameter** | **Mean difference** | **95% confidence interval** | | **Bootstrap P value** |
| --- | --- | --- | --- | --- |
| SOFA + PCT | 0.3435 | [0.2702, 0.4398] | ＜0.001 | |
| SOFA + METTL7B | 0.3423 | [0.2443, 0.4406] | ＜0.001 | |
| PCT + METTL7B | 0.3273 | [0.2256, 0.4265] | ＜0.001 | |
| SOFA + PCT + METTL7B | 0.3749 | [0.2702, 0.4749] | ＜0.001 | |

Figure S1: Mean difference and 95% confidence interval

Figure S2: Comparison of AUC values for each indicator combination

Figure S3: Association between baseline METTL7B levels and ΔSOFA including patients who died before day 7.

Note: Only patients surviving to day 7 were included in this analysis (n = 56); patients who died before day 7 were not included because day-7 SOFA scores were unavailable. The central line in the box represents the median, the box represents the IQR, and the whiskers show the range.
